# Belly Dancer's Dyskinesia or Functional Movement Disorder: Involuntary Abdominal Movements in a Pediatric Patient

**DOI:** 10.7759/cureus.88291

**Published:** 2025-07-19

**Authors:** Michael J Sinnott, Carlos Suanes, Eddy Hernandez Cuesta, Skyler Brandfon, Andrea Maggioni

**Affiliations:** 1 Medicine, Florida International University, Herbert Wertheim College of Medicine, Miami, USA; 2 Pediatrics, Nicklaus Children's Hospital, Miami, USA; 3 Hospital Services, Nicklaus Children's Hospital, Miami, USA

**Keywords:** abdominal movements, abdominal spasms, belly dancer's dyskinesia (bdd), functional movement disorder, pediatric

## Abstract

Belly dancer's dyskinesia (BDD) is a rare movement disorder marked by involuntary, rhythmic, undulating movements of the abdominal wall, often mistaken for gastrointestinal or psychiatric conditions. We describe the case of a 13-year-old girl with a seven-year history of abdominal spasms that stopped during sleep and resumed on waking. The episodes were accompanied by loud "whooshing" sounds from the abdomen. Her medical history included primary ciliary dyskinesia and a ventricular septal defect. She had never received medication for her symptoms. On examination, the right side of her upper body twisted in sync with the abdominal movements, which lessened when she was distracted. An extensive workup including brain and spine magnetic resonance imaging (MRI), electroencephalogram (EEG), echocardiogram, and metabolic panels was unremarkable. With no clear underlying cause, a diagnosis of BDD was made. Further evaluation by a psychiatrist showed grunting and sighing, as well as trunk and shoulder flexion associated with movements suggesting a functional movement disorder (FMD). The patient was started on clonidine 0.1 mg for symptomatic relief and was referred for cognitive behavioral therapy (CBT). Although she was lost to follow-up, this case highlights the clinical overlap between BDD and FMD and the importance of considering it in patients with unexplained abdominal movements.

## Introduction

Belly dancer’s dyskinesia (BDD) is a rare movement disorder first described by Iliceto et al. in 1990 [1]. It is characterized by involuntary, rhythmic, and often writhing contractions of the abdominal musculature that mimic the undulating movements of a belly dancer [2]. These movements may occur spontaneously, fluctuate with distraction or sleep, and can cause physical discomfort and psychosocial implications [2]. Although BDD is typically benign, its presentation can be particularly disruptive in pediatric patients.

There are fewer than 60 cases documented in the literature to date, the majority of which involve adults and show a predominance in females [2]. The etiology of BDD is often unclear, though proposed causes include central or peripheral neurological mechanisms, phrenic nerve irritation, metabolic disturbances, medication side effects, and functional neurological disorders [2-4]. Diagnostic evaluation is often inconclusive, and no standardized guidelines exist for diagnosis or management, making clinical recognition and treatment challenging.

We present a unique case of BDD in a 13-year-old female with a complex history of congenital heart disease and primary ciliary dyskinesia. Her prolonged, progressively worsening abdominal spasms ultimately led to significant physical symptoms and social distress. She was originally diagnosed with BDD by the neurology team during hospitalization but was later diagnosed with functional movement disorder (FMD) by psychiatry. Her final diagnosis remains unclear due to overlap of clinical features and loss of follow-up. Similarities between BDD and FMD make the disorders challenging to distinguish. This case underscores the need for increased awareness of BDD in pediatric populations as a possible diagnosis and highlights the importance of a thorough, multidisciplinary approach in evaluating movement disorders with overlapping functional and organic features.

## Case presentation

A 13-year-old female with a medical history of primary ciliary dyskinesia, ventricular septal defect, and a seven-year history of acute on chronic abdominal spasms presented to the emergency department with worsening abdominal spasms. Her spasms primarily involved the abdominal muscles but have recently worsened and now involve the upper body causing falls, rib pain, abdominal pain, and social impacts such as bullying. She also reported loud abdominal sounds during episodes, described as a “whooshing” noise of fluid.

She noted that the abdominal spasms lasted seconds to hours and had increasingly worsened as she aged. The episodes ceased during sleep and resumed upon awakening, with progressive worsening throughout the day. She dealt with a lot of school-related stress and sleep disturbances that she noted were due to constantly moving and being bullied at school. She had tried non-pharmacological treatments, including heat, ice, massage, and Epsom salt baths, without relief. No pharmacological therapy had ever been initiated. Multiple doctors were seen in the past, including a neurologist who suspected Tourette syndrome, a second neurologist who suspected dystonia, and a gastroenterologist who suspected a possible food allergy and suggested endoscopic evaluation. All her previous physicians were lost to follow-up due to frequent relocations.

Her physical exam showed mild tenderness over the ribs and occasional right-sided upper body twisting with abdominal contractions. Movement while supine showed wave-like contractions of the entire abdomen from the thorax down to the pelvis with associated peristaltic sound. The remainder of her physical exam was unremarkable. Laboratory findings, including comprehensive metabolic panel, thyroid function tests, vitamin B12, magnesium, antinuclear antibody (ANA), and human chorionic gonadotropin (hCG), were within normal limits (Table 1). Cardiac evaluation with electrocardiogram (ECG) and echocardiography were unremarkable. Magnetic resonance imaging (MRI) of the brain and cervical, thoracic, and lumbar spine was normal. During her hospitalization, the patient was initially diagnosed with BDD by the neurology team. 

**Table 1:** Comprehensive Laboratory Results

Test	Result	Reference Range
Sodium (Na)	138 mmol/L	135–145 mmol/L
Potassium (K)	4 mmol/L	3.5–5.0 mmol/L
Chloride (Cl)	104 mmol/L	98–106 mmol/L
Carbon Dioxide (CO₂)	21.6 mmol/L	22–29 mmol/L
Glucose	90 mg/dL	70–99 mg/dL
Blood Urea Nitrogen (BUN)	9 mg/dL	7–20 mg/dL
Creatinine (Cr)	0.59 mg/dL	0.6–1.3 mg/dL
Calcium (Ca)	10.1 mg/dL	8.5–10.5 mg/dL
Albumin	4.9 g/dL	3.5–5.5 g/dL
Alanine Aminotransferase (ALT)	14 U/L	7–56 U/L
Aspartate Aminotransferase (AST)	27 U/L	10–40 U/L
Magnesium (Mg)	1.8 mg/dL	1.7–2.2 mg/dL
Lipase	138 U/L	0–160 U/L
Antinuclear Antibody (ANA)	Negative	Negative
Vitamin B12	349 pg/mL	200–900 pg/mL
Free T4 (fT4)	1.2 ng/dL	0.8–1.8 ng/dL
Triiodothyronine (T3)	123 ng/dL	80–200 ng/dL
Thyroid Stimulating Hormone (TSH)	0.98 μIU/mL	0.4–4.0 μIU/mL
Thyroid Peroxidase Antibody	Positive	Negative
Human Chorionic Gonadotropin (hCG)	Negative	Negative

Later in the admission, psychiatry evaluated the patient and noted that the spasms were decreased when the patient was distracted or engaged in conversation. Additionally, prior videos provided by her parents revealed episodes of grunting along with trunk and shoulder flexion. These findings led to a new possible diagnosis of FMD. Electromyography (EMG) was withheld due to these new findings. The patient was started on clonidine 0.1 mg nightly for symptomatic management. She was discharged home with outpatient follow-up arranged for cognitive behavioral therapy (CBT) but did not return for subsequent care.

## Discussion

BDD is a rare movement disorder first discovered in 1990 when Iliceto et al. noticed a series of rhythmic and repetitive involuntary abdominal wall movements resembling those of a belly dancer [1]. A review of 59 cases showed that approximately 61% were female between the ages of seven and 85 [2]. Most cases were found to be bilateral and were caused by medications, surgical procedures, pregnancies, B12 deficiency, or idiopathic in origin [2]. BDD is characterized by involuntary, rhythmic, and repetitive abdominal muscle contractions. The contractions are slow and writhing and may involve the upper and lower abdominal regions [2]. BDD can present suddenly or gradually, with some patients complaining of abdominal pain or discomfort that accompanies the abdominal movements [2]. In our patient, the abdominal spasms were slow writhing and involved the upper body, raising suspicion for BDD. Due to the limited number of cases, age-specific differences and long-term outcomes remain poorly understood, especially in pediatric patients.

The pathophysiology behind BDD is not well understood but there are several hypotheses. One mechanism suggests the spinal inhibitory interneurons cause abnormal excitability and involuntary abdominal contractions as demonstrated by the presence of a central spinal pattern generator, rhythmic motor patterns, on EMG [3]. A second hypothesis suggests that irritation of the phrenic nerve may cause diaphragmatic movement, leading to abdominal contractions [2]. BDD may be related to other movement disorders such as myoclonus, which involves sudden, brief involuntary muscle contractions [4]. Although this presentation may appear similar to that of diaphragmatic myoclonus, BDD movements are slow with writhing features as seen in this case. Additionally, the absence of identifiable triggers such as medication or spinal pathology made idiopathic or central causes more plausible. 

When diagnosing BDD, tests should be ordered based on clinical suspicion given the patient's history. Diagnostic workup may include EMG, videofluoroscopy, MRI of the brain and spinal cord, and CT of the chest but often yield inconclusive results, which was seen in this patient [2]. Treatment options for BDD should focus on symptom management. Interventions may include oral diazepam, ultrasound-guided botulinum toxin A injections, or transcutaneous electrical nerve stimulation [5-7]. Imaging of the patient using MRI of the brain and spine was unremarkable. Pharmacologic treatment was not initiated due to diagnostic uncertainty and emerging signs pointing to a functional etiology.

FMD is a subtype of a functional neurological disorder that is characterized by abnormal movements such as tremors, dystonia, myoclonus, or weakness that are inconsistent with neurological diseases and not explained by structural pathology [8]. Positive clinical features such as variability, distractibility, and internal inconsistency are key diagnostic hallmarks that help make the diagnosis rather than being a diagnosis of exclusion [8]. FMD has a neuropsychiatric pathophysiology caused by abnormal brain network connectivity between the limbic and motor regions [9]. Psychological stressors may also exist but are not necessary for the diagnosis. 

BDD and FMD can both present with bizarre variable movements. The key to differentiating the two lies in identifying positive clinical features of FMD such as distractibility, variability, incongruence, and abrupt onset. Organic BDD is more likely to be persistent and non-modifiable by distraction and may originate from lesions to the premotor cortex, primary motor cortex, basal ganglia, spine, peripheral nerves, or abdominal muscles [2]. Because there is no definitive test for either disorder, a thorough history and exam for positive signs of FMD are necessary to distinguish the two. In this case, the presence of distractibility, symptom variability, and psychosocial stressors such as school-related anxiety and frequent relocations aligned more closely with the features of FMD than classical BDD. 

Treatment for FMD should be multidisciplinary through the use of physical therapy, CBT, and individualized psychiatric support [10]. Physical therapy allows for motor retraining and redirecting attention away from abnormal movement patterns improving outcomes such as quality of life and mobility. CBT addresses maladaptive thoughts and behaviors, which reduces symptom severity, improving overall function [10]. While some patients improve with treatment, long-term outcomes can be variable and many patients have persistent symptoms [10]. The patient was started on clonidine which is not specific to treating FMD but was useful due to its anxiolytic and autonomic calming abilities. It is uncertain whether their symptoms would have responded better to behavioral interventions rather than pharmacological due to lack of longitudinal follow-up.

## Conclusions

BDD is a rare and poorly understood movement disorder with very few cases in the literature. Despite multiple proposed mechanisms, the exact pathophysiology remains unclear, making diagnosis challenging. Although the initial diagnosis in this case was BDD, the evolution of findings such as distractibility, variability, and psychosocial stressors led to the diagnosis of FMD. The overlapping features of BDD and FMD increase the complexity of diagnosis and underscore the importance of taking a thorough history, physical exam, and multidisciplinary collaboration. This case contributes to the limited pediatric literature on both conditions and emphasizes the need for awareness, particularly in children with unexplained abdominal movements. Further research is required to better understand these diseases and target optimal diagnostic and treatment strategies.

## References

[1] Iliceto G, Thompson PD, Day BL, Rothwell JC, Lees AJ, Marsden CD (1990). Diaphragmatic flutter, the moving umbilicus syndrome, and "belly dancer's" dyskinesia. Mov Disord.

[2] Rissardo JP, Vora NM, Tariq I, Batra V, Caprara AL (2024). Unraveling belly dancer's dyskinesia and other puzzling diagnostic contortions: a narrative literature review. Brain Circ.

[3] Tamburin S, Idone D, Zanette G (2007). Belly dancer's myoclonus and chronic abdominal pain: pain-related dysinhibition of a spinal cord central pattern generator?. Parkinsonism Relat Disord.

[4] Aggarwal A, Thompson PD (2011). Unusual focal dyskinesias. Handb Clin Neurol.

[5] Alshubaili A, Abou-Al-Shaar H, Santhamoorthy P, Attia H, Bohlega S (2016). Ultrasound-guided botulinum toxin A injection in the treatment of belly dancer's dyskinesia. BMC Neurol.

[6] Ossella C, Dell'Omo F, Zanetti E, Pestalozza IF, Galizia P, Tripodi S (2021). Belly dancer syndrome: an unusual cause of abdominal pain. Pediatr Emerg Care.

[7] Linazasoro G, Van Blercom N, Lasa A, Fernández JM, Aranzábal I (2005). Etiological and therapeutical observations in a case of belly dancer's dyskinesia. Mov Disord.

[8] Hallett M, Aybek S, Dworetzky BA, McWhirter L, Staab JP, Stone J (2022). Functional neurological disorder: new subtypes and shared mechanisms. Lancet Neurol.

[9] Baizabal-Carvallo JF, Hallett M, Jankovic J (2019). Pathogenesis and pathophysiology of functional (psychogenic) movement disorders. Neurobiol Dis.

[10] LaFaver K (2020). Treatment of functional movement disorders. Neurol Clin.

